# Both LDL and HDL particle concentrations associate positively with an increased risk of developing microvascular complications in patients with type 2 diabetes: lost protection by HDL (Zodiac-63)

**DOI:** 10.1186/s12933-023-01909-1

**Published:** 2023-07-06

**Authors:** Arno R. Bourgonje, Margery A. Connelly, Harry van Goor, Peter R. van Dijk, Robin P. F. Dullaart

**Affiliations:** 1 Department of Gastroenterology and Hepatology, University of Groningen, University Medical Center Groningen, P.O. Box 30.001, 9700 RB Groningen, The Netherlands; 2 The Henry D. Janowitz Division of Gastroenterology, Department of Medicine, Icahn School of Medicine at Mount Sinai, New York, NY USA; 3 Labcorp, Morrisville, NC USA; 4 Department of Pathology and Medical Biology, University of Groningen, University Medical Center Groningen, Groningen, The Netherlands; 5 Department of Internal Medicine, Division of Endocrinology, University of Groningen, University Medical Center Groningen, Groningen, The Netherlands

**Keywords:** Type 2 diabetes, Microvascular complications, Lipoproteins, HDL, LDL

## Abstract

**Background:**

Triglyceride-rich lipoproteins (TRL) and low-density lipoproteins (LDL) are associated positively whereas high-density lipoproteins (HDL) are associated inversely with the development of new-onset type 2 diabetes (T2D). Here we studied potential associations between these lipoprotein particle concentrations and the risk of developing microvascular complications in patients with established T2D.

**Methods:**

Lipoprotein particle concentrations (TRLP, LDLP, and HDLP) were determined in 278 patients with T2D participating in a primary care-based longitudinal cohort study (Zwolle Outpatient Diabetes project Integrating Available Care [ZODIAC] study) leveraging the Vantera nuclear magnetic resonance (NMR) platform using the LP4 algorithm. Associations between lipoprotein particles and incident microvascular complications (nephropathy, neuropathy, and retinopathy) were assessed using Cox proportional hazards regression models.

**Results:**

In total, 136 patients had microvascular complications at baseline. During a median follow-up of 3.2 years, 49 (34.5%) of 142 patients without microvascular complications at baseline developed new-onset microvascular complications. In multivariable Cox proportional hazards regression analyses, both total LDLP and HDLP concentrations, but not total TRLP concentrations, were positively associated with an increased risk of developing any microvascular complications after adjustment for potential confounding factors, including age, sex, disease duration, HbA1c levels, history of macrovascular complications, and statin use (adjusted hazard ratio [HR] per 1 SD increment: 1.70 [95% CI 1.24–2.34], *P* < 0.001 and 1.63 [95% CI 1.19–2.23], *P* = 0.002, respectively). When analyzing each microvascular complication individually, total LDLP concentrations were positively associated with retinopathy (adjusted HR 3.35, 95% CI 1.35–8.30, *P* = 0.009) and nephropathy (adjusted HR 2.13, 95% CI 1.27–3.35, *P* = 0.004), and total HDLP concentrations with neuropathy (adjusted HR 1.77, 95% CI 1.15–2.70, *P* = 0.009). No significant associations were observed for lipoprotein particle subfractions.

**Conclusions:**

Total lipoprotein particle concentrations of both LDL and HDL associate positively with an increased risk of developing microvascular complications in T2D. We propose that the protective role of HDL on the development of microvascular complications may be lost in established T2D.

**Supplementary Information:**

The online version contains supplementary material available at 10.1186/s12933-023-01909-1.

## Background

Individuals with type 2 diabetes (T2D) commonly exhibit alterations in circulating lipoprotein particles and their subfractions, captured as increased levels of triglyceride-rich apolipoprotein B (apoB)-containing lipoproteins, e.g. very large very low-density lipoproteins (VLDL) and small low-density lipoproteins (LDL), and decreased levels of high-density lipoproteins (HDL) [1–10]. Altered levels of circulating lipoprotein particles associate with impaired glucose tolerance and new-onset T2D, suggesting an early role in its pathogenesis [11–16]. Moreover, previous studies have also shown cross-sectional and longitudinal associations of elevated LDL-cholesterol with albuminuria in adult patients with type 1 diabetes, T2D, with nephropathy in children with type 1 diabetes (T1D) as well as with albuminuria in non-diabetic individuals [17–21], Furthermore, modest and inconsistent changes in lipoprotein levels were found in diabetic retinopathy [21, 22], and a prospective association of raised triglycerides with the development of diabetic neuropathy [23, 24]. Few studies so far have evaluated the associations between different types of lipoprotein particles, their subfractions, and the development of microvascular complications in established T2D [15, 16, 25, 26].

Notably, methods based on nuclear magnetic resonance (NMR) spectroscopy have been developed that facilitate large scale and more in-depth characterization of lipoprotein particles and their subfractions [27]. Accumulating evidence suggests that measurements of lipoprotein particles may have merit in predicting the onset of T2D. Indeed, multiple population-based studies have used such methods to examine associations between triglyceride-rich lipoprotein (TRL), LDL, and HDL particle concentrations (TRLP, LDLP and HDLP) and their subfractions with the risk of new-onset T2D [28–33]. For example, TRL and LDL particle concentrations and characteristics (subfractions and sizes) have recently been associated with incident T2D in a large population-based cohort of almost 5,000 individuals, as well as with changes in β-cell function independent of insulin resistance [28]. Notably, however, associations of comprehensive NMR-determined lipoprotein particle concentrations and subfractions with the risk of developing microvascular complications in established T2D have, to the best of our knowledge, not yet been explored.

Here we hypothesized that lipoprotein particle concentrations and their subfractions may be of potential utility as biomarkers for the development of microvascular complications in individuals with T2D. In this study, we aimed to determine associations between lipoprotein particles and their subfractions with the risk of developing microvascular complications in patients with T2D through leveraging an NMR-based platform. To do so, we measured lipoprotein particles in patients participating in a primary care-based longitudinal cohort study of individuals with T2D.

## Methods

### Study population

This study featured samples and data from the Zwolle Outpatient Diabetes project Integrating Available Care (ZODIAC) study, which is a longitudinal observational cohort study recruiting primary care-treated patients with established T2D in the Zwolle region of the Netherlands. For this study, plasma samples obtained at baseline were analyzed from individuals with T2D of whom data on prevalence and follow-up development of microvascular complications (i.e. either diabetic nephropathy, neuropathy, or retinopathy) was available. After exclusion of four patients with missing follow-up data, the evaluable cohort consisted of 278 patients of whom 136 had microvascular complications at baseline. These patients were excluded from the follow-up analysis leaving 142 patients for the longitudinal analysis. This study was approved by the Institutional Review Board (IRB) of the Isala Hospital Zwolle, the Netherlands (IRB reference nos. 03.0316 and 07.0335). All patients provided written informed consent for study participation.

### Study outcomes and definitions

Primary outcome of this study was the development of microvascular complications including diabetic nephropathy, neuropathy and retinopathy. Development of complications was assessed annually using a standardized protocol. Diabetic nephropathy was defined as the presence of at least two consecutive measurements of elevated albuminuria (which was determined as albumin-to-creatinine ratio > 3.5 mg/mmol for women and > 2.5 mg/mmol for men) or one measurement of elevated albuminuria in case the patient was receiving treatment with angiotensin-converting enzyme-inhibitors or angiotensin-II-receptor antagonists. The development of diabetic neuropathy was defined as two or more (out of three) errors on foot sensibility tests on at least one foot when using a 5.07 Semmes–Weinstein monofilament. The development of diabetic retinopathy was established through fundus imaging using a retinal camera and performed by trained ophthalmologists.

### Measurements of lipoprotein particles

Ethylenediaminetetraacetic acid (EDTA) plasma samples at baseline were collected and stored at −80ºC until further analysis. The lipoprotein particle concentrations and related parameters were determined by NMR LipoProfile^®^ testing (Labcorp, Morrisville, NC, USA), as described previously [27–29]. NMR spectra were determined using the LP4 algorithm embedded in an optimized version of the NMR LipoProfile test [27]. TRLP (subfractions: very large, large, medium, small, and very small), LDLP (subfractions: large, medium, and small) and HDLP (subfractions: small, medium, and large) were measured leveraging the conventional deconvolution method and the amplitudes of their corresponding lipid methyl group NMR signals that are spectroscopically distinct [27, 29]. Total concentrations of TRLP, LDLP and HDLP were calculated as the sum of their individual subfractions. Precision (%CV) for the lipoprotein classes were (within-lab): TRLP:10.0–5.4%, LDLP:3.1–2.0% and HDLP:1.3–1.2% for low to high concentration pools. Estimated ranges of particle diameters for the TRL and LDL particles were as follows: very large TRLP, 90–240 nm; large TRLP, 50–89 nm; medium TRLP, 37–49 nm; small TRLP, 30–36 nm; very small TRLP, 24–29 nm; large LDLP, 21.5–23 nm; medium LDLP, 20.5–21.4 nm; and small LDLP, 19–20.4 nm. Estimated ranges of particle diameter for the HDL subclasses were: small HDL, 7.4 to 8.0 nm; medium HDL, 8.1 to 9.5 nm; large HDL, 9.6 to 13 nm [34, 35]. Precision (%CV) for the lipoprotein subclasses varied as follows (within-lab): TRLP subclasses: 9.9–18.3%, LDLP subclasses:3.8–19.6% and HDLP: 3.8–10.9%.

Triglycerides (TG), total cholesterol (TC), LDL-cholesterol (LDL-c), HDL-cholesterol (HDL-c), and Apolipoprotein B (ApoB) concentrations were calculated following equations derived from lipoprotein measures that were analyzed on a Vantera Clinical NMR Analyzer platform using partial least squares (PLS) regression models [36]. As described, model performance yielded coefficients of variation (CVs) between 3.7 and 6.0% for these lipids and (apo)lipoprotein measures [33]. Correlation coefficients with chemically measured concentrations ranged from R = 0.980 to R = 0.997, and within-lab CVs ranged from 1.0 to 3.6% [36]. Apolipoprotein A-1 (apoA-1) concentrations were determined using linear regression of the lipoprotein HDL subclass signal areas against serum apolipoprotein levels measured chemically in a large reference range study population (n = 698). Precision (%CV) for apoA-I: 2.2 to 1.7% for low to high concentration pools and the correlation coefficient for the comparison with chemically measured apoA-I: R = 0.930 (n = 6,831).

### Statistical analysis

Baseline descriptive statistics of the study population were presented as means ± standard deviation (SD) or as medians with interquartile ranges (IQR). Categorical variables were given as numbers with corresponding percentages (%). Baseline characteristics were presented for the total study population and according to the (non-)development of microvascular complications. Differences in baseline characteristics between patients with microvascular complications at baseline, those who developed and did not not develop microvascular complications during follow-up were tested using one-way analysis of variance (ANOVA) or Kruskal–Wallis tests for continuous variables followed by post-hoc testing (independent sample *t*-tests or Mann–Whitney *U*-tests, respectively), and chi-square tests for categorical variables. Univariable correlations were analyzed by Spearman correlation analysis. Univariable and multivariable Cox proportional hazards regression analyses were performed to study associations between lipoprotein particles and the risk of development of microvascular complications, of which results were expressed as hazard ratios (HRs) with corresponding 95% confidence intervals. Lipoprotein particles were standardized resulting in HRs expressed as per 1-SD increment or decrement. Proportionality of hazards was checked to ascertain absence of violating assumptions. Statistical analysis was performed using SPSS Statistics 28.0 software (SPSS Inc., Chicago, IL, USA). Two-tailed *P* < 0.05 were considered statistically significant.

## Results

### Baseline cohort characteristics

Baseline demographic, clinical and laboratory characteristics of the total study population are presented in Table 1. Of the 136 patients with microvascular complications at baseline, 74 (54.4%) had nephropathy, 61 (44.9%) had retinopathy and 63 (46.3%) had neuropathy alone or in combination. Patients without microvascular complications at baseline who did not develop microvascular complications during follow-up were younger, had a shorter diabetes duration, a lower HbA1c and a history of macrovascular complications less frequently. Besides diet and lifestyle modifications, patients were treated with metformin, sulfonylurea and insulin; other glucose-lowering drugs were not used. Patients without microvascular complications at baseline who did not develop microvascular complications during follow-up were treated with diet alone more frequently and with metformin and/or insulin less frequently. There were no significant differences in statin use between the groups.

**Table 1:** Baseline demographic, clinical and laboratory data of patients with microvascular complications at baseline, patients without microvascular complications at baseline but developing microvascular complications during follow-up and patients without microvascular complications not developing microvascular complications during follow-up

	Microvascular complications at baseline (*n* = 136)	No microvascular complications at baseline but occurring during follow-up (*n* = 49)	No microvascular complications at baseline and not occurring during follow-up (*n* = 93)	Overall *P*-value	*P*-value*
Age (years)	66.9 ± 9.7	67.0 ± 10.3	61.5 ± 10.4	< 0.001	< 0.001
Sex				0.419	0.189
Male, *n* (%)	68 (50.0)	25 (51.0)	39 (41.9)		
Female, *n* (%)	68 (50.0)	24 (49.0)	54 (58.1)		
BMI (kg/m^2^)	27.9 [25.2–31.2]	30.1 [25.5–33.9]	28.6 [24.7–31.5]	0.253	0.463
Disease duration (years)	5.0 [2.1–10.0]	3.0 [1.4–6.0]	2.6 [1.3–5.0]	< 0.001	< 0.001
Systolic blood pressure (mmHg)	150 [135–160]	150 [140–163]	145 [135–164]	0.854	0.576
Current smoking, *n* (%)	29 (21.8)	6 (12.2)	16 (17.2)	0.311	0.683
History of macrovascular complications, *n* (%)	63 (46.3)	15 (30.6)	20 (21.5)	< 0.001	< 0.001
Diabetes therapy at baseline				< 0.001	< 0.001
Diet alone, *n* (%)	11 (8.1)	8 (16.3)	24 (25.8)		
Metformin, *n* (%)	31 (31.6)	9 (28.1)	10 (20.0)		
SU-derivatives, *n* (%)	49 (50.0)	19 (59.4)	31 (62.0)		
Insulin, *n* (%)	20 (14.7)	3 (6.1)	1 (1.1)		
Both, *n* (%)	12 (8.8)	4 (8.2)	3 (3.2)		
Statin use, *n* (%)	28 (20.9)	6 (12.2)	16 (17.2)	0.414	0.764
HbA1c (%)	6.9 [6.3–8.0]	7.0 [6.3–8.2]	6.5 [5.9–7.3]	0.002	< 0.001
HbA1c (mmol/mol)	51.9 [45.5–63.9]	53.0 [45.4–66.1]	47.5 [41.0–56.3]	0.002	< 0.001
eGFR (ml/min/1.73m^2^)	69.5 [57.5–93.8]	75.0 [60.0–88.0]	77.7 [59.2–93.7]	0.406	0.264
Total TRLP (nmol/L)	166 [120–228]	172 [128–205]	141 [100–209]	0.089	0.028
Very large TRLP (nmol/L)	0.30 [0.10–0.73]	0.50 [0.10–1.50]	0.20 [0.00–0.90]	0.202	0.147
Large TRLP (nmol/L)	6.30 [2.80–12.2]	6.80 [3.70–13.4]	5.50 [2.60–12.2]	0.704	0.632
Medium TLRP (nmol/L)	25.1 [15.8–35.8]	23.9 [16.5–34.4]	24.2 [14.0–38.1]	0.993	0.907
Small TRLP (nmol/L)	29.8 [11.6–46.0]	33.7 [11.4–49.4]	27.6 [7.5–44.5]	0.583	0.379
Very small TLRP (nmol/L)	93.1 [51.3–156.3]	97.2 [47.1–144.2]	76.8 [35.5–134.7]	0.257	0.105
Total LDLP (nmol/L)	1750 [1429–2071]	1813 [1480–2086]	1666 [1394–1935]	0.131	0.062
Large LDLP (nmol/L)	436 [278–714]	464 [253–747]	491 [284–780]	0.689	0.404
Medium LDLP (nmol/L)	546 [131–872]	527 [62–935]	317 [49–686]	0.087	0.028
Small LDLP (nmol/L)	640 [439–975]	717 [480–1000]	691 [421–955]	0.776	0.864
Total HDLP (nmol/L)	21.1 [19.7–23.3]	21.1 [19.6–23.4]	20.9 [19.2–23.1]	0.775	0.509
Large HDLP (µmol/L)	0.80 [0.30–1.50]	0.50 [0.20–1.50]	0.60 [0.25–1.40]	0.345	0.479
Medium HDLP (µmol/L)	4.95 [3.98–6.83]	4.90 [3.90–6.45]	5.00 [4.10–6.50]	0.890	0.631
Small HDLP (µmol/L)	15.1 [13.1–16.7]	15.1 [12.6–16.9]	14.8 [12.6–16.9]	0.829	0.547
TRL size	54.6 [47.2–61.2]	55.8 [48.6–62.5]	54.2 [48.5–61.6]	0.805	0.786
LDL size	21.0 [20.8–21.4]	21.0 [20.7–21.4]	21.1 [20.7–21.5]	0.895	0.988
HDL size	8.7 [8.5–8.9]	8.6 [8.4–9.0]	8.6 [8.4–8.9]	0.555	0.398
Triglycerides (mg/dL)	195 [146–265]	192 [155–271]	173 [135–242]	0.390	0.181
Total cholesterol (mg/dL)	200 [179–229]	201 [182–237]	196 [166–219]	0.148	0.055
LDL-cholesterol (mg/dL)	114 [93–137]	120 [93–140]	111 [89–129]	0.210	0.088
HDL-cholesterol (mg/dL)	44 [38–52]	43 [38–53]	44 [37–51]	0.866	0.667
ApoB (mg/dL)	101 [87–125]	105 [86.5–127]	98.0 [82.0–114]	0.083	0.033
ApoA-1 (mg/dL)	124 [112–141]	123 [115–140]	125 [112–135]	0.853	0.593

Data are presented as means ± SD, medians with IQR. Categorical variables are given in numbers with corresponding percentages (%)

*eGFR* estimated glomerular filtration rate, *HbA1c* hemoglobin A1c, *HDLP* high-density lipoprotein particles, *LDLP* low-density lipoprotein particles, *TRLP* triglyceride-rich lipoprotein particles

^*^P-values for comparisons between patients without microvascular complications at baseline nor during follow-up *versus* patients either with microvascular complications at baseline or developing microvascular complications during follow-up

The total TRLP concentration was lowest in those patients without microvascular complications at baseline who did not develop microvascular complications during follow-up, with trends for lower TRL subfractions except for medium TRLP. The total LDLP concentration and the medium LDLP concentration tended to be lower in patients without microvascular complications at baseline who did not develop microvascular complications during follow-up. Neither the total HDLP concentration, nor HDL subfractions were higher in patients without microvascular complications at baseline who did not develop microvascular complications during follow-up.

TG, TC and LDL-c tended to be lower and apoB was significantly lower in patients without microvascular complications at baseline who did not develop microvascular complications during follow-up, but there were no between-group differences in HDL-cholesterol and apoA1.

In the total cohort, TRLP was correlated with triglycerides (ρ = 0.30, *P* < 0.001), LDLP was correlated with LDL-cholesterol (ρ = 0.94, *P* < 0.001) and apoB (ρ = 0.97, *P* < 0.001) and HDLP was correlated with HDL-cholesterol (ρ = 0.60, *P* < 0.001) and apoA-I (ρ = 0.82, *P* < 0.001).

### Lipoprotein particle concentrations and the development of microvascular complications

Over a median follow-up of 3.2 years [IQR: 2.9–3.4 years], 49 of 142 individuals (34.5%) developed at least one microvascular complication. Of these, 19 patients developed nephropathy, 29 neuropathy, and 10 patients retinopathy, either alone or in combination. Cox proportional hazards regression analyses revealed a close to significant association between total TRLP concentrations and the risk of developing microvascular complications (*Model 1*, HR per 1-SD increment 1.31, 95% CI 1.00–1.71, *P* = 0.051), while statistically significant associations were observed for total LDLP (*Model 1*, HR 1.66, 95% CI 1.23–2.24, *P* = 0.001) and HDLP concentrations (*Model 1*, HR 1.44, 95% CI 1.11–1.87, *P* = 0.007) (Table 2). Upon adjustment for age and sex, the association between total TRLP concentrations and the risk of developing microvascular complications was statistically significant (*Model 2*, HR 1.45, 95% CI 1.07–1.94, *P* = 0.015), and associations for total LDLP and HDLP concentrations remained statistically significant (*Model 2*, HR 1.75, 95% CI: 1.29–2.38, *P* < 0.001 and HR 1.58, 95% CI 1.19–2.10, *P* = 0.001, respectively). After additional adjustment for disease duration, HbA1c levels, history of macrovascular complications, and use of statins, the association between total TRLP concentrations and the risk of developing microvascular complications was not significant (*Model 4*, HR 1.27, 95% CI 0.93–1.75, *P* = 0.135), while associations for total LDLP and HDLP concentrations remained significantly associated (*Model 4*, HR 1.70, 95% CI 1.24–2.34, *P* < 0.001 and HR 1.63, 95% CI 1.19–2.23, *P* = 0.002).

**Table 2:** Cox proportional hazards regression analyses for associations between lipoprotein fractions and the risk of developing microvascular complications in patients with type 2 diabetes

	Model	Total microvascular complications		*P*-value
		HR	95% CI	
Total TRLP concentration	1	1.31	1.00–1.71	0.051
	2	**1.45**	1.07–1.94	**0.015**
	3	1.27	0.93–1.74	0.130
	4	1.27	0.93–1.75	0.135
Total LDLP concentration	1	**1.66**	1.23–2.24	**0.001**
	2	**1.75**	1.29–2.38	** < 0.001**
	3	**1.72**	1.26–2.36	** < 0.001**
	4	**1.70**	1.24–2.34	** < 0.001**
Total HDLP concentration	1	**1.44**	1.11–1.87	**0.007**
	2	**1.58**	1.19–2.10	**0.001**
	3	**1.55**	1.15–2.09	**0.004**
	4	**1.63**	1.19–2.23	**0.002**

HRs are expressed per 1-SD increment. Model 1: crude. Model 2: model 1, plus age and sex. Model 3: model 2, plus adjustment for disease duration, HbA1c and history of macrovascular complications. Model 4: model 3, with adjustment for statin use

Statistically significant associations are indicated in bold

When analyzing the development of diabetic neuropathy, nephropathy and retinopathy as individual outcomes, total TRLP concentrations appeared to be significantly associated with the risk of developing retinopathy (*Model 1*, HR 2.10, 95% CI 1.24–3.58], *P* = 0.006), albeit statistical significance disappeared after adjustment for potential confounding factors (*Model 4*, HR 1.77, 95% CI 0.93–3.37], *P* = 0.084, Table 3). Similarly, total LDLP concentrations were significantly associated with the risk of developing diabetic retinopathy, which remained after adjustment for the same potential confounding factors (*Model 4*, HR 3.35, 95% CI 1.35–8.30, *P* = 0.009). Total LDLP were also significantly associated with the risk of developing nephropathy (*Model 4*, HR 2.13, 95% CI 1.27–3.35, *P* = 0.004). Unlike LDLP concentrations, total HDLP concentrations were not significantly associated with either diabetic nephropathy or retinopathy in fully adjusted analyses, but were significantly associated with the risk of developing neuropathy, also after adjustment for potential confounding factors (*Model 4*, HR 1.77, 95% CI 1.15–2.70, *P* = 0.009). Except for medium LDLP, none of the lipoprotein subfractions (for TRLP, LDLP, and HDLP) were significantly associated with the risk of developing microvascular complications in crude analyses, neither in combination (Additional file 1: Table S1) nor with respect to individual complications (data not shown). In addition, there were no associations of TRL, LDL or HDL size with incident microvascular complications (Additional file 1: Table S1).

**Table 3:** Cox proportional hazards regression analyses for associations between lipoprotein fractions and the risk of developing diabetic nephropathy, neuropathy and retinopathy in patients with type 2 diabetes

	Model	Nephropathy			Neuropathy			Retinopathy		
		HR	95% CI	*P*-value	HR	95% CI	*P*-value	HR	95% CI	*P*-value
Total TRLP concentration	1	1.23	0.78–1.93	0.375	1.25	0.88–1.78	0.217	**2.10**	1.24–3.58	**0.006**
	2	1.36	0.83–2.22	0.218	1.42	0.95–2.12	0.088	**2.34**	1.34–4.07	**0.003**
	3	1.11	0.64–1.92	0.704	1.29	0.85–1.95	0.231	1.76	0.93–3.31	0.081
	4	1.05	0.60–1.84	0.867	1.29	0.85–1.97	0.230	1.77	0.93–3.37	0.084
Total LDLP concentration	1	**1.71**	1.05–2.80	**0.032**	1.32	0.89–1.96	0.170	**2.93**	1.42–6.06	**0.004**
	2	**1.83**	1.11–3.00	**0.017**	1.42	0.95–2.11	0.089	**3.24**	1.54–6.79	**0.002**
	3	**2.12**	1.26–3.58	**0.005**	1.32	0.89–1.97	0.164	**3.31**	1.33–8.24	**0.010**
	4	**2.13**	1.27–3.55	**0.004**	1.28	0.86–1.91	0.229	**3.35**	1.35–8.30	**0.009**
Total HDLP concentration	1	1.18	0.76–1.85	0.461	**1.45**	1.03–2.03	**0.031**	**2.00**	1.06–3.79	**0.032**
	2	1.25	0.79–1.98	0.349	**1.70**	1.17–2.47	**0.005**	**1.91**	1.00–3.63	**0.049**
	3	1.31	0.80–2.14	0.280	**1.60**	1.08–2.38	**0.020**	1.77	0.68–4.58	0.239
	4	1.31	0.78–2.23	0.309	**1.77**	1.15–2.70	**0.009**	1.78	0.69–4.59	0.232

HRs are expressed per 1-SD increment. Model 1: crude. Model 2: model 1, plus age and sex. Model 3: model 2, plus adjustment for disease duration, HbA1c and history of macrovascular complications. Model 4: model 3, with adjustment for statin use

Statistically significant associations are indicated in bold

Finally, when analyzing derived concentrations of TG, LDL-cholesterol and HDL-cholesterol in relation to the risk of developing microvascular complications, only LDL- and HDL-cholesterol were significantly associated, whereas triglyceride concentrations were not (Table 4).

**Table 4:** Cox proportional hazards regression analyses for associations between standard lipid measures (triglycerides, LDL-cholesterol and HDL-cholesterol) and the risk of developing microvascular complications in patients with type 2 diabetes

	Model	Total microvascular complications		*P*-value
		HR	95% CI	
Triglycerides	1	1.08	0.87–1.35	0.485
	2	1.17	0.92–1.48	0.203
	3	1.06	0.83–1.37	0.634
	4	1.05	0.81–1.36	0.702
LDL-cholesterol	1	1.54	1.16–2.05	**0.003**
	2	1.65	1.24–2.20	** < 0.001**
	3	1.68	1.25–2.26	** < 0.001**
	4	1.66	1.23–2.23	** < 0.001**
HDL-cholesterol	1	1.40	1.06–1.86	**0.019**
	2	1.41	1.07–1.86	**0.014**
	3	1.44	1.08–1.92	**0.014**
	4	1.45	1.08–1.94	**0.013**

HRs are expressed per 1-SD increment. Model 1: crude. Model 2: model 1, plus age and sex. Model 3: model 2, plus adjustment for disease duration, HbA1c and history of macrovascular complications. Model 4: model 3, with adjustment for statin use

Statistically significant associations are indicated in bold

## Discussion

We have demonstrated that within a primary care-based cohort of individuals with established T2D and a relatively short diabetes duration (median < 3 years), higher circulating concentrations of both total LDL and HDL particles are associated with an increased risk of development of microvascular complications, even after adjustment for relevant covariates. When analyzing microvascular complications individually, total LDL particle concentrations were significantly associated with an increased risk of developing diabetic nephropathy and retinopathy, whereas total HDL particle concentrations were associated with an increased risk of developing diabetic neuropathy, both after full adjustment for potential confounding factors. Total TRL particle concentrations were associated with the development of any microvascular complication and diabetic retinopathy only in partly adjusted analyses. Except for medium LDL particles, lipoprotein subfractions of TRL, LDL, and HDL particles were not significantly associated with the risk of developing microvascular complications in crude analyses, neither as a composite outcome nor with individual complications. However, point estimates of the hazard ratios of associations with TRL, LDL and HDL particles were always above 1.0 potentially suggesting adverse effects of these lipoprotein subfractions with new-onset microvascular complications. Collectively, our results suggest a relevant involvement of both LDL and HDL particle concentrations in the development of microvascular complications in T2D, with a paradoxical association for HDL that is postulated to take on a pro-inflammatory role in the context of established T2D.

The total TRL and LDL particle concentrations as found in the present study among T2D patients who had microvascular complications at baseline and among patients who did and did not develop microvascular complications are higher than those in non-diabetic participants from the PREVEND cohort study who did not develop T2D during follow-up. In our previous report using the same NMR platform and also applying the LP4 algorithm, the total TRL particle and LDL particle concentrations amounted to 147 nmol/L and 1467 nmol/L, respectively [29]. Furthermore, the total HDL particle concentration was modestly lower in the currently studied T2D cohort compared to that previously reported in non-diabetic subjects [29]. These comparisons corroborate the impact of the diabetic state on apoB-containing lipoprotein elevations with some reductions in HDL particles.

Of note, in the present study, measurements of TG, LDL-c and HDL-c, were derived from NMR-based equations. In secondary analyses with these conventional lipid- and lipoprotein measures, the association of triglycerides with incident microvascular complications did not reach significance, but we observed a positive association with LDL-c and HDL-c both in crude as well as in fully adjusted analysis, congruent with our main analysis on LDL and HDL particles.

Our findings regarding the association of total LDL particle concentrations with incident microvascular complications, in particular nephropathy and retinopathy, are in line with other studies demonstrating that LDL-cholesterol is elevated in T2D individuals with microalbuminuria [19] and retinopathy [22], although we could not pinpoint this association to small LDL particles, which are known to be more susceptible to oxidative modification and glycation [37, 38]. In comparison, it was previously observed in the population-based Prevention of Renal and Vascular End-Stage Disease (PREVEND) cohort study that there is a positive association between albuminuria and apoB-containing lipoproteins (LDL cholesterol, non-HDL cholesterol, triglycerides and apolipoprotein-B) [20]. In that report, albuminuria was found to interact with apoB-containing lipoproteins on incident cardiovascular disease (CVD), suggesting common pathogenic pathways between albuminuria and apoB-containing lipoproteins in the development of cardiovascular disease (CVD) [20]. In addition, we observed positive associations with total TRL particle concentrations, in particular retinopathy, but these associations did not reach significance in fully adjusted analysis, likely due to a lack of statistical power with only 10 individuals developing retinopathy alone or in conjunction with other microvascular complications.

The most salient and novel finding of the present study is the paradoxical positive association of total HDL particle concentrations with incident microvascular complications. This association was significant with neuropathy, but hazards with other complications were also greater than zero. To our knowledge, the only other report so far in T2D showing that HDL, in this case HDL-cholesterol, is positively instead of inversely associated with comorbidities is a large-scale cross-sectional study from China with osteoporosis as outcome [39]. In women with T1D, it has been demonstrated that very high HDL cholesterol levels greater than 80 mg/dL are positively associated with incident atherosclerotic CVD [40]. HDL is able to promote cell-derived cholesterol efflux, and has anti-oxidative, anti-inflammatory and anti-thrombotic properties, all of which are considered to represent protective functionalities [40–44]. Notably, it has been proposed that under circumstances of enhanced chronic inflammation, HDL is dysfunctional and may even become proinflammatory [41]. Indeed, early in vitro studies have shown that administration of LDL-derived oxidized phospholipids may render HDL proinflammatory [45]. Against this background, we surmise that HDL function is impaired under hyperglycemic circumstances. Evidence from this supposition comes from studies showing attenuated HDL anti-oxidative and anti-inflammatory functions in T2D coinciding with decreased activity of the anti-oxidative enzyme paraoxonase-1 (PON-1) [46, 47], and reduced HDL antithrombotic capacity coinciding with microvascular diabetic complications and lower lipid content in the smallest HDL subfraction (measured using another NMR platform) in a genetically mixed South African population of T2D patients with a White Dutch or South Asian background [48]. Moreover, the athero-protective effect of HDL-cholesterol efflux may be lost in a population enriched with individuals with (pre)diabetes [49]. On the other hand, HDL-cholesterol efflux as well as the anti-inflammatory capacity of HDL was found to be maintained despite an increased triglyceride/cholesteryl ester ratio and alterations in the lipidome in HDL from T2D patients [50]. With incident atherosclerotic CVD as the outcome, support of dysfunctional HDL as pro-atherogenic lipid biomarker comes from observational findings in several population-based studies showing that the inverse association of HDL-c with CVD development is lost at very high HDL-c concentrations [51, 52]. In the PREVEND cohort study, we demonstrated that CVD risk is increased in individuals with concurrently high levels of the chronic inflammation marker, high sensitivity C-reactive protein (hs-CRP), and HDL-cholesterol [53], with a plausible role of decreased PON-1 activity [54]. Also, the highest apoB-adjusted CVD incidence was observed in PREVEND participants with concomitant microalbuminuria and a high apoA-1/HDL particle ratio, reflecting the apoA-1 content per HDL particle [55]. Finally, we have recently reported that plasma levels GlycA, an NMR-measured glycosylation marker of five major acute phase reactants, which is closely correlated with hs-CRP, is associated with incident microvascular complications in the ZODIAC cohort [56]. Notably, GlycA is positively related to the total HDL particle concentration, a preferred lipid biomarker over HDL-cholesterol in assessing CVD risk [57], and inversely with HDL-cholesterol efflux capacity in the multi-ethnic Dallas Heart Study, involving over 2,600 participants [58]. In view of these reports, our current findings regarding the positive association of total HDL particle concentrations with incident microvascular complications, consent with the hypothesis that dysfunctional HDL may play a pathogenic role in such diabetic complications, conceivably due to enhanced chronic inflammation. This hypothesis needs to be tested in future HDL function studies in the context of diabetic microvascular complication development.

Several strengths and limitations of this study need to be acknowledged. A strength of this study pertains to the well-documented nature of this primary care-based cohort of patients with T2D, consisting of deeply phenotyped individuals in a prospective longitudinal study design. Following these aspects, we were able to reliably assess relationships between lipoprotein particles and the occurrence of developing microvascular complications in T2D, while being able to control for a variety of relevant covariates. Furthermore, in this study we characterized lipoprotein particle profiles using NMR spectroscopy, which allowed for more comprehensive measurements of lipoprotein concentrations and their subfractions. A limitation of the present longitudinal analysis is the rather small population suitable for longitudinal analysis due to the presence of microvascular complications of about half of the total study cohort at baseline. Another limitation inherent to the longitudinal, observational study design and correlative nature of this study is that we could not fully exclude the possibility of reverse causation. Second, since the majority of individuals included in this cohort were inhabitants of a specific geographical area in the Netherlands, this may preclude generalizability of our findings to other more ethnically diverse cohorts of patients with T2D. Finally, there was no use of SGLT2 and GLP-1 receptor agonists in this cohort which was established before the introduction of these agents in the clinic.

## Conclusions

Although our analyses were performed in a rather small cohort of T2D patients, this study suggests that both high LDL and HDL particle concentrations, as proxies of lipoprotein metabolic disturbances, are associated with an increased risk of developing microvascular complications in individuals with established T2D. These findings support the potential merit of measurement of lipoprotein particle concentrations as clinically relevant lipid biomarkers for the development of microvascular complications in T2D. Future studies are warranted to further validate our findings as well as to determine the potential therapeutic amenability of elevated lipoprotein particles with the goal of attenuating the risk of developing microvascular complications.

## Supplementary Information

**Additional file 1: Table S1**. Cox proportional hazards regression analyses for associations between lipoprotein subfractions and -sizes and the risk of developing any microvascular complications in patients with type 2 diabetes.

## Data Availability

The datasets used and analyzed during the current study are available from the corresponding author on reasonable request.

## Abbreviations

ANOVA: Analysis of variance
ApoA-1: Apolipoprotein A-1
ApoB: Apolipoprotein B
BMI: Body mass index
CV: Coefficient of variation
CVD: Cardiovascular disease
CI: Confidence interval
EDTA: Ethylenediaminetetraacetic acid
GLP-1: Glucagon-like peptide 1
HDL: High-density lipoproteins
Hs-CRP: High sensitivity C-reactive protein
HR: Hazard ratio
HDL-c: HDL-cholesterol
IRB: Institutional Review Board
LDL: Low-density lipoproteins
LDL-c: LDL-cholesterol
NMR: Nuclear magnetic resonance
PON-1: Paraoxonase-1
PLS: Partial least squares
PREVEND: Prevention of renal and vascular end-stage disease
SGLT2: Sodium-glucose cotransporter-2
SD: Standard deviation
TC: Total cholesterol
TG: Triglycerides
TRL: Triglyceride-rich lipoproteins
T2D: Type 2 diabetes
ZODIAC: Zwolle Outpatient Diabetes project Integrating Available Care
