# Supplementary material for: Both LDL and HDL particle concentrations associate positively with an increased risk of developing microvascular complications in patients with type 2 diabetes: lost protection by HDL (Zodiac-63)
Source: Cardiovasc Diabetol. 2023 Jul 6;22:169. doi: 10.1186/s12933-023-01909-1 (PMC10327395; doi:10.1186/s12933-023-01909-1)
Supplement: Supplementary file 1 — Additional file 1: Table S1. Cox proportional hazards regression analyses for associations between lipoprotein subfractions and -sizes and the risk of developing any microvascular complications in patients with type 2 diabetes. [file 12933_2023_1909_MOESM1_ESM.docx]

**Additional file**

**Additional file Table 1**. Cox proportional hazards regression analyses for associations between lipoprotein subfractions and -sizes and the risk of developing any microvascular complications in patients with type 2 diabetes.

|  | **Incident microvascular complications** | |  |
| --- | --- | --- | --- |
|  | HR | 95% CI | *P*-value |
| **TRLP subfractions** |  |  |  |
| Very large TRLP | 1.15 | 0.93-1.40 | 0.194 |
| Large TRLP | 1.06 | 0.82-1.37 | 0.671 |
| Medium TLRP | 1.05 | 0.79-1.40 | 0.724 |
| Small TRLP | 1.10 | 0.88-1.37 | 0.411 |
| Very small TLRP | 1.25 | 0.93-1.67 | 0.140 |
| **LDLP subfractions** |  |  |  |
| Large LDLP | 1.10 | 0.82-1.47 | 0.521 |
| Medium LDLP | 1.30 | 1.01-1.67 | **0.045** |
| Small LDLP | 1.05 | 0.77-1.42 | 0.762 |
| **HDLP subfractions** |  |  |  |
| Large HDLP | 1.24 | 0.93-1.63 | 0.140 |
| Medium HDLP | 1.27 | 0.96-1.67 | 0.091 |
| Small HDLP | 1.27 | 0.96-1.68 | 0.091 |
| **Lipoprotein sizes** |  |  |  |
| TRL size | 0.96 | 0.71-1.28 | 0.764 |
| LDL size | 1.01 | 0.75-1.36 | 0.949 |
| HDL size | 1.10 | 0.82-1.48 | 0.510 |

HRs are expressed per 1-SD increment. Crude analyses are shown. Statistically significant associations are indicated in **bold**.
